# A Rare Case of Intravenous Amiodarone Toxicity

**DOI:** 10.7759/cureus.27958

**Published:** 2022-08-13

**Authors:** Ana Lopes dos Santos, Margarida Lagarto, Cláudio Gouveia

**Affiliations:** 1 Internal Medicine, Hospital São Francisco Xavier, Lisbon, PRT; 2 Oncology, Hospital São Francisco Xavier, Lisbon, PRT

**Keywords:** benzyl alcohol, polysorbate 80, adverse drug reaction, refractory hypotension, acute hepatoxicity, intravenous amiodarone

## Abstract

Amiodarone is a highly effective treatment for life-threatening supraventricular and ventricular arrhythmias, namely in the setting of acutely decompensated heart failure. However, it could be associated with several serious adverse effects both in long-term oral therapy and in short-term use of intravenous (IV) preparation, including shock and liver injury. We report an unusual case of life-threatening refractory hypotension associated with acute hepatitis and renal failure a few hours after initiation of IV amiodarone. A 70-year-old man was admitted to the emergency department (ED) with dyspnea, chest discomfort, and a non-productive cough. Physical examination and complementary diagnostic tests helped diagnose acutely decompensated heart failure due to atrial fibrillation (AF) with a rapid ventricular response, and IV amiodarone was started. A few hours after initiating this drug, the patient developed hypotension with the need for inotropic therapy, acute elevation of amino transaminases, and renal failure. Renal function and liver transaminases returned to baseline after discontinuing amiodarone. A Roussel Uclaf Causality Assessment Method (RUCAM) score of 5 identifies our patient`s acute hepatitis as a possible adverse drug reaction. Refractory hypotension and liver injury with acute hepatitis after a short-term IV amiodarone therapy are extremely rare with few previously reported cases. Therefore, it is very important to perform continuous hemodynamic monitoring of the patient and liver function monitorization during short-term IV administration of this drug because these complications can be potentially fatal. A high index of suspicion is the key to functional organic recovery.

## Introduction

Amiodarone is a class III antiarrhythmic drug commonly used to treat a wide spectrum of ventricular and supraventricular tachyarrhythmias with high effectiveness. It has the advantage of being given either orally or intravenously [1-3]. Chronic oral administration has a well-documented toxicity profile and involves many organ systems, including pulmonary fibrosis, thyroid dysfunction, and liver injury with an asymptomatic rise of liver transaminases in approximately 25% of patients and symptomatic hepatotoxicity in less than 3% of long-term users [2-4]. Major adverse effects of oral amiodarone are related to an accumulation of the drug and its active metabolite (mono-N-desethylamiodarone (MDEA)) in fatty tissues and, sometimes, result in a poor prognosis because of their very long plasma half-life [1,3,5]. Its adverse effects result in the cessation of medication in 10-15% of patients [5]. IV amiodarone is typically used as a short-term therapy for the management of potentially life-threatening arrhythmias. More common adverse effects following IV administration include injection site reaction, diaphoresis, flushing, sinus bradycardia, atrioventricular block, and hypotension, potentially life-threatening and refractory. In rare cases, occurring annually in approximately 1% of those receiving this drug, IV amiodarone can cause acute hepatoxicity that presents from mild asymptomatic increase in serum transaminases to hepatitis and fulminant hepatic failure [4,5]. Thus, although rare, it is important to be familiar with these complications that can be fatal. We report an unusual case of life-threatening refractory hypotension associated with acute hepatitis and renal failure a few hours after initiation of intravenous amiodarone that illustrates the importance of a high index of suspicion.

## Case presentation

A 70-year-old man presented to the emergency department (ED) with dyspnea, shortness of breath with minimal exertion, chest discomfort, and non-productive cough without fever. His symptoms had gradually worsened over the prior week and his chest discomfort had increased for the past four hours before he presented to the ED.

He was previously diagnosed to have chronic heart failure, atrial fibrillation (AF), and chronic obstructive pulmonary disease (COPD) due to tobacco smoking. There was no history of alcohol intake or illicit drug use. No medication adherence. No known drug or any other allergies. Echocardiography performed six months ago showed heart failure with mildly reduced left ventricular ejection fraction.

On examination, his consciousness was clear and he did not have breathing difficulty at rest with no oxygen supply, peripheric oxygen saturation was 99%, radial pulse rate was 158 beats per minute (bpm), which was irregular, upper-arm blood pressure was 145/103mmHg, and his body temperature was 36.5ºC. Cardiac auscultation confirmed irregular heart sounds without any murmur, pulmonary auscultation with bibasilar rales, and prolonged expiratory time. Bilateral low limb edema up to the knees was present with Godet ++.

The electrocardiogram (ECG) at presentation showed AF with a rapid ventricular response (cardiac frequency 160bpm) with no ischemic changes. Chest X-ray with emphysematous pattern with slight signs of congestion without pleural effusion or pulmonary consolidation. Blood tests showed normal complete blood count, creatinine 1.28mg/dL (reference range 0.7-1.2 mg/dL), all electrolytes within normal limits, and C-reactive protein 1.20 mg/dL (reference range <0.5 mg/dL). Total bilirubin 0.98 mg/dL (reference range <1.4 mg/dL), alkaline phosphatase (FA) 60 U/L (reference range 40-130 U/L), gamma-glutamyltransferase (GGT) 20 U/L (reference range 10-71 U/L), aspartate aminotransferase (AST) 32 U/L (reference range <40 U/L), alanine aminotransferase (ALT) 38 U/L (reference range <41 U/L), and lactate dehydrogenase (LDH) 110 U/L (reference range 120-246 U/L). The international normalized ratio (INR) was 1.5. N-terminal-pro hormone brain natriuretic peptide (NT-proBNP) was mildly elevated (17,937 pg/mL). High sensitive troponin at the time of presentation was 18ng/L with a variation less than 10ng/L after three hours.

We admitted diagnosis of acutely decompensated heart failure due to AF with a rapid ventricular response. The patient was initially treated with IV metoprolol until 15mg, which did not have any effect on his cardiac frequency. Amiodarone was commenced as an IV loading dose of 300mg following a continuous IV infusion of 600mg at a maximum rate of 72mg per hour. After 18 hours, the patient became confused and diaphoretic and his systolic blood pressure started to drop below 60mmHg; central cyanosis with mottling score 3 was present. Lactate was very high, 9.2mmol/L. Amiodarone infusion was stopped. A test dose of 500ml of isotonic fluid did not have any effect on blood pressure and an inotropic IV infusion of noradrenaline 13mcg/min was started. There were no features of an acute anaphylactic reaction (no signs of rash, itching, or eosinophilia) and no changes in the ST segment or T-waves in the ECG. All subsequent troponin levels were within normal range. Echocardiography showed left ventricular ejection fraction of 45% with no changes in segmental kinetics. Pulmonary embolism or acute aortic dissection were excluded by computed tomography angiography (CTA) of the chest. Infectious origin was excluded with a negative microbiological screening.

The patient was stabilized in the ED and then transferred to the intensive care unit (ICU). During these first 18 hours after admission in the hospital, he developed acute hepatic and renal failure with metabolic acidemia. Blood tests showed total bilirubin 4.83 mg/dL, conjugated bilirubin 1.46 mg/dL, AST 10310 U/L, ALT 5056 U/L, GGT 17 U/L, FA 62 U/L, LDH 12436 U/L, ammonia 115 mg/dL, INR 3.6, albumin 3.3g/dL, and creatinine 2.78mg/dL. At this point, we reviewed all medications given and serologic study results were negative for chronic viral hepatitis and other liver diseases. Abdominal echography showed homogeneous hepatomegaly without other alterations, namely ascites or splenomegaly.

**Figure 1:**
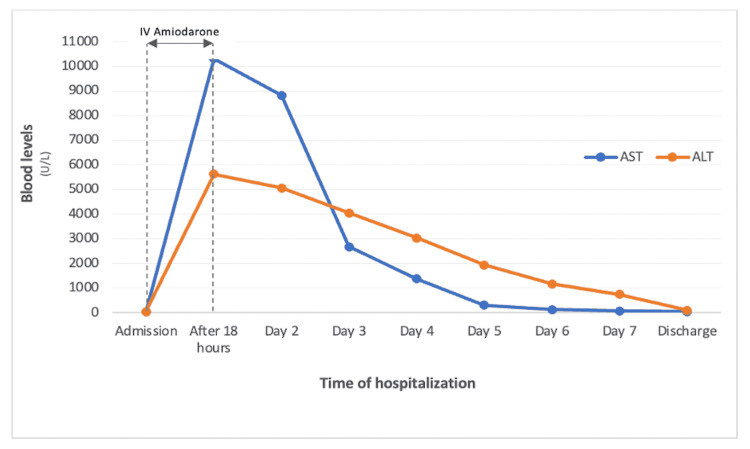
Time evolution of the blood transaminase levels AST: aspartate aminotransferase; ALT: alanine aminotransferase

After 24 hours of cessation of IV amiodarone, he had globally favorable evolution with the suspension of inotropic infusion, correction of metabolic acidemia, consistent reduction of serum lactate, and progressive normalization of renal and liver parameters (Table 1 and Figure 1).

**Table 1:** Liver and renal blood test analysis AST: aspartate aminotransferase; ALT: alanine aminotransferase; GGT: gamma-glutamyltransferase; FA: alkaline phosphatase; INR: international normalized ratio

Days of hospitalization	AST (U/L)	ALT (U/L)	GGT (U/L)	FA (U/L)	Total bilirubin (mg/dL)	INR	Creatinine (mg/dL)	Ammonia (mg/dL)	Albumin (mg/dL)
Admission	32	38	20	60	0.98	1.5	1.28	115	3.3
After 18 hours	10310	5625	17	62	4.83	3.6	2.78		
Day 2	8817	5056	36	66	3.02	3.3	2.45		
Day 3	2679	4046	32	68	2.78	2.6	1.87		
Day 4	1372	3032	46	67	2.5	2.5	1.90		
Day 5	299	1929	56	75	2.0	2.1	1.72		
Day 6	123	1167	55	70	1.4	2.0	1.56		
Day 7	71	742	55	68	1.1	1.6	1.3	52	3.6
Discharge	36	102	32	60	0.82	1.3	1.42		

Previously, oral bisoprolol and digoxin were commenced with heart rate control, amiodarone was not re-introduced because amiodarone-induced toxicity was suspected. A Roussel Uclaf Causality Assessment Method (RUCAM) score of 5 identifies our patient`s acute hepatitis as a possible adverse drug reaction. The probability score and the progressive clinical and analytical recovery after discontinuation of amiodarone led us to assume that the drug was the causal agent. An adverse reaction to amiodarone was assumed and an adverse reaction statement was made in conjunction with the pharmaceutical services. The patient was discharged from the hospital after 16 days.

## Discussion

Amiodarone is an iodinated benzofuran derivative and is one of the most effective antiarrhythmic drugs available. It acts by interference with potassium, sodium, and calcium channels and it also has beta-blocking effects. However, it is generally considered as a class III antiarrhythmic agent according to the Vaughan-Williams classification [6]. IV amiodarone is widely used in EDs to control life-saving arrhythmias in the setting of heart failure due to this lack of negative inotropic effects and high efficacy in cardiac arrhythmias resistant to other treatments [5]. Thus, in this case, we previously used metoprolol for frequency control but without success, and so we chose to start amiodarone in a patient with no known drug allergies. However, approximately 18 hours after the start of the IV infusion of amiodarone, the patient developed life-threatening hypotension and needed to start inotropes. At the same time, after analytical re-evaluation, renal failure and a very significant increase in aminotransferases were observed, compatible with acute hepatitis, with a spontaneous increase in INR value. Cases of acute hepatoxicity after short-term use of IV amiodarone typically occur within the first 24 hours after the infusion is started. These attend with a predominant increase in transaminases, with values greater than 10 times the upper limit of normality, without cholestasis, and with improvement after the withdrawal of amiodarone. Less commonly, renal impairment or coagulation disorders are present [4].

The mechanism of IV amiodarone-associated life-threatening hypotension and hepatotoxicity is not clear and became controversial, but it is unlikely due to amiodarone itself. Amiodarone is not stable in aqueous solutions and, therefore, must be dissolved in a solvent, such as a mixture of polysorbate 80 (polyoxenethylated sorbitan ester) and a small amount of benzyl alcohol [1,5,7]. Those molecules exert hemodynamic effects. Polysorbate 80 has a potent vasodilator and negative inotropic effects, unlike amiodarone itself which has low inotropic action [7,8]. Benzyl alcohol is an aromatic alcohol used as a bacteriostatic and solvent and has been responsible for metabolic acidosis, respiratory insufficiency, seizures, and hypotension leading to cardiovascular collapse in newborns [7]. Furthermore, polysorbate 80 has been implicated in the E-ferol syndrome, characterized by hepatosplenomegaly, cholestatic jaundice, renal failure, and thrombocytopenia, a cause of hepatotoxicity in infants who present clinical and histological similarities to those found in cases of liver toxicity due to IV amiodarone [5,8,9].

Some authors ascribe the responsibility for hemodynamic instability and liver toxicity to the diluents because eliminating these solvents by the oral route demonstrated the safe use of oral amiodarone, even after acute hepatitis and severe hypotension, in several studies [1,7,9].

The hypothesis that the two excipients, benzyl alcohol and polysorbate 80, precipitated the occurrence of the shock seems plausible in a patient who had chronic heart failure exacerbated by arrhythmia. However, unfortunately, we were unable to measure the serum level of the two excipients and the plasma concentration of amiodarone and DEA to confirm this hypothesis. There were several case reports on acute hypotension with IV amiodarone use thought to be due to an anaphylactic reaction [6]. However, the drug anaphylaxis has shown other associated symptoms or signs of an allergic reaction such as angioedema or urticaria with hypotension not seen in this case. Other causes of shock were excluded with repeat ECGs and troponin levels, CTA of the chest, and a microbiological screening.

On the other hand, abnormal liver function is common in ICUs and it is very important to differentiate the cause. The etiologies consist of viral hepatitis, ischemia, autoimmune disease, and medication [10]. Drug-induced liver injury is a diagnosis of exclusion, even in cases where the liver injury is likely to be drug-related. We have excluded viral, autoimmune, and neoplastic etiologies as causes of acute hepatitis, by the assessment of image exams and extended analytical panel. We performed probability scores of adverse drug reaction, namely the RUCAM score, that helps to assess the likelihood of drug-induced liver injury. Causality assessment by the updated RUCAM requires prior evaluation of liver injury criteria and its pattern in each suspected case [11]. A RUCAM score of 5 identifies our patient`s acute hepatitis as a possible adverse drug reaction but this relatively low score may be related to the fact that we did not expose the patient to the IV drug again. This probability score and the progressive clinical and analytical recovery after discontinuation of amiodarone led us to assume that the drug was the causal agent. However, hepatic ischemia caused by hemodynamic instability (also an adverse effect of IV amiodarone) and poor cardiac output can also play a role in amiodarone-induced hepatotoxicity (liver ischemia with superimposed direct drug toxicity). We believe that in susceptible elderly patients, even the standard IV amiodarone dose may cause slight hypotension, especially in the setting of heart failure, contributing to hepatic injury.

It was chosen to give oral bisoprolol and digoxin for long heart rate control. However, since the mechanisms of oral and IV amiodarone toxicity differ, it can be administrated with safety, if necessary, provided that liver and renal parameters are closely monitored [3,4,8].

## Conclusions

IV amiodarone is widely used in EDs to control life-saving arrhythmias because of its high efficacy in cardiac arrythmias resistant to other treatments. However, the use of current IV amiodarone formulations can lead to the occurrence of refractory hypotension as well as the occurrence of severe acute hepatitis with eventual liver failure. Therefore, although this is rare, it remains important because of the popularity of IV amiodarone use in the treatment of severe arrythmias. Thus, physicians should be aware to these adverse effects which, although rare, can be fatal, and a high index of suspicion is the key to the functional organic recovery. The patient should be under continuous hemodynamic monitoring with close surveillance and transaminases should be monitored regularly during IV administration of amiodarone. Iatrogenic drug causes should always be considered when inpatients deteriorate.

Since the solvents used in the IV formulation are implicated in these types of adverse effects, the pharmaceutical industry should be encouraged to develop new amiodarone formulations with other types of solvents. On the other hand, it would be important to carry out studies for the development of antidotes for amiodarone-induced hypotension until better intravenous amiodarone preparations are made available to clinicians.
